# Supplementary material for: How octopuses use and recruit additional arms to find and manipulate visually hidden items
Source: Biol Open. 2025 Jul 10;14(7):bio062011. doi: 10.1242/bio.062011 (PMC12309893; doi:10.1242/bio.062011)
Supplement: Supplementary information [file biolipen-14-062011-s1.pdf]

Table S1a. The initial arm inserted into the dome in the first approach of each trial (n = 117 trials, initial approaches), recorded per octopus.

	Arm							
Octopus	R1	R2	R3	R4	L1	L2	L3	L4
A	3	3	0	2	2	1	0	1
B	2	3	0	1	3	2	0	1
C	1	1	1	0	3	3	0	0
D	2	4	0	0	3	2	0	1
E	4	1	0	0	3	3	1	0
F	3	1	1	1	1	4	0	1
G	0	1	0	0	1	1	0	1
H	2	0	0	0	2	0	0	0
I	1	1	0	0	1	0	0	1
J	3	0	0	0	0	0	1	0
K	3	0	0	0	1	1	1	2
L	0	0	4	0	1	0	1	2
M	1	4	0	0	2	0	1	0
N	0	3	1	1	0	1	0	2

Table S1b. The initial arm inserted into the dome in additional approaches during each trial (*n* = 513 additional approaches), recorded per octopus.

	Arm							
Octopus	R1	R2	R3	R4	L1	L2	L3	L4
A	7	7	4	4	12	7	3	6
B	8	14	5	10	11	14	8	9
C	5	5	2	1	6	3	12	2
D	9	10	4	6	7	10	2	5
E	7	1	7	13	2	8	6	0
F	4	4	4	10	2	3	7	11
G	1	1	2	2	2	2	2	4
H	2	0	0	1	1	2	1	1
I	1	2	5	3	3	4	2	2
J	0	0	1	1	0	1	2	0
K	7	7	0	5	3	3	5	5
L	3	6	7	5	3	7	10	8
M	2	4	3	0	5	3	6	2
N	5	6	4	6	4	4	8	9

Biology Open • Supplementary information

Table S2. Total observed occurrences of arms used for exploring the dome's interior in each trial (n = 117), recorded per octopus and separated by the item presented. Grey cells indicate the octopus was not presented with that item. Zeros indicate true zero occurrences.

	Frozen Crab (n = 38 trials)								3D Rock Shape (n = 20 trials)							
Octopus	R1	R2	R3	R4	L1	L2	L3	L4	R1	R2	R3	R4	L1	L2	L3	L4
A	10	6	9	12	9	8	5	9	1	1	2	1	3	2	2	3
B	12	15	6	8	13	8	5	5	4	1	2	3	7	7	6	4
C	4	4	1	0	3	3	1	1	2	1	1	0	4	5	3	1
D	11	10	4	6	11	9	4	4	6	4	2	1	3	5	3	3
E	3	8	7	10	1	9	10	8	1	2	2	3	1	3	1	1
F	8	7	7	5	5	9	9	9	2	4	2	1	3	3	3	4
G																
H																
I																
J																
K	6	6	0	5	8	9	3	6	2	3	1	2	2	1	3	2
L	6	8	16	9	9	12	12	9	5	2	2	3	1	3	4	6
M	6	10	7	5	7	6	9	9	2	4	0	2	2	4	1	1
N	7	8	9	9	5	5	7	13	3	6	5	2	3	4	1	4

	Agarose Disc (n = 19 trials)								3D Crab Shape (n = 20 trials)							
Octopus	R1	R2	R3	R4	L1	L2	L3	L4	R1	R2	R3	R4	L1	L2	L3	L4
A	4	6	1	2	4	4	1	1	5	3	1	0	4	0	0	2
B	3	7	4	11	8	12	8	4	3	1	2	1	5	1	1	1
C	2	0	0	0	3	4	1	1	3	8	7	5	3	2	9	5
D	5	3	3	4	2	9	5	3	7	10	5	6	3	7	2	3
E	4	4	3	5	4	2	0	5	10	5	2	0	3	3	4	4
F	3	2	3	2	4	3	4	6	5	0	3	2	6	5	4	3
G									1	2	3	2	4	2	1	3
H									4	4	0	1	5	2	2	2
I									2	6	6	5	7	8	6	4
J									5	5	1	5	2	7	3	1
K	7	6	1	2	2	5	4	3								
L	3	4	4	8	3	4	7	2								
M	0	4	2	3	3	2	6	2								
N	2	2	2	4	1	4	4	3								

	Live Crab (n = 20 trials)							
Octopus	R1	R2	R3	R4	L1	L2	L3	L4
A	7	1	0	4	6	3	1	1
B	3	4	5	5	5	8	6	6
C	5	7	8	0	5	3	4	4
D	5	1	3	2	3	4	0	2
E	5	6	1	4	2	1	1	3
F	0	1	0	1	0	2	0	1
G	3	3	3	2	1	2	4	3
H	2	2	0	2	2	1	2	0
I	2	1	2	5	5	9	5	1
J	2	3	4	1	3	5	8	5
K								
L								
M								
N								

Table S3a. Total number of explorations inside of the dome prior to the initial contact of the item in each trial (*n* = 117), recorded per octopus.

Octopus	Initial contact made immediately (no prior explorations)	Number of explorations prior to initial contact													
		1	2	3	4	5	6	7	8	9	10	11	12	13	14
A	5	3	2	0	0	0	1	0	0	0	0	0	1	0	0
B	3	2	3	3	1	0	0	0	0	0	0	0	0	0	0
C	1	2	2	3	0	0	1	0	0	0	0	0	0	0	0
D	5	1	2	1	1	0	1	0	0	0	0	0	0	1	0
E	3	4	3	1	0	1	0	0	0	0	0	0	0	0	0
F	3	4	3	2	0	0	0	0	0	0	0	0	0	0	0
G	2	2	0	0	0	0	0	0	0	0	0	0	0	0	0
H	1	1	1	0	0	0	0	0	0	0	1	0	0	0	0
I	2	1	0	1	0	0	0	0	0	0	0	0	0	0	0
J	2	1	0	0	0	0	0	1	0	0	0	0	0	0	0
K	3	2	1	1	0	1	0	0	0	0	0	0	0	0	0
L	3	1	2	0	1	0	0	0	0	0	0	0	0	0	1
M	2	2	2	1	1	0	0	0	0	0	0	0	0	0	0
N	4	0	1	2	0	0	0	0	0	0	0	1	0	0	0

Table S3b. Exploratory behaviors following item extraction from the dome (*n* = 70 trials in which the item was extracted), recorded in total occurrences per octopus.

	Number of additional approaches to the dome										Maximum number of arms used to explore inside of the dome in each approach							
Octopus	0	1	2	3	4	5	6	7	8	9	1	2	3	4	5	6	7	8
A	1	2	1	0	1	0	0	1	0	0	1	1	1	1	1	0	0	0
B	1	0	0	1	2	0	0	2	0	1	2	1	3	0	1	0	0	0
C	2	0	0	0	0	1	1	0	0	1	0	0	2	0	1	0	0	0
D	1	0	1	4	1	0	0	1	0	0	0	3	2	1	1	0	0	0
E	1	1	2	0	1	1	1	0	0	0	1	4	1	0	0	0	0	0
F	3	0	1	1	2	0	0	0	0	0	1	1	2	0	1	0	0	0
G	1	0	0	1	0	0	1	1	0	0	0	3	0	0	0	0	0	0
H	2	0	0	1	0	0	0	0	0	0	0	1	0	0	0	0	0	0
I	1	0	0	0	0	0	1	1	0	0	0	2	0	0	0	0	0	0
J	2	1	0	0	1	0	0	0	0	0	0	1	0	1	0	0	0	0
K	1	0	1	0	0	1	1	0	0	0	0	1	1	1	1	0	0	0
L	0	1	0	1	1	1	0	0	0	0	0	1	1	1	1	0	0	0
M	0	0	1	1	2	0	0	0	0	0	1	1	1	1	0	0	0	0
N	0	0	0	1	1	1	0	1	0	0	0	1	2	1	0	0	0	0

Table S4. Total observed occurrences of arms used for contacting the item inside of the dome in each trial (*n* = 117), recorded per octopus and separated by the item presented. Grey cells indicate the octopus was not presented with that item. Zeros indicate true zero occurrences.

	Frozen Crab (n = 38 trials)								3D Rock Shape (n = 20 trials)							
Octopus	R1	R2	R3	R4	L1	L2	L3	L4	R1	R2	R3	R4	L1	L2	L3	L4
A	0	2	1	2	0	2	0	1	5	3	2	0	2	4	1	2
B	3	0	0	0	3	1	2	3	3	6	0	3	6	2	2	3
C	3	2	1	1	3	2	2	1	1	1	0	0	1	0	2	1
D	3	2	2	1	2	4	2	1	2	3	1	0	2	3	1	1
E	1	5	3	2	2	1	4	1	2	0	2	2	0	3	0	0
F	3	1	2	5	4	5	2	3	0	3	0	3	1	3	4	3
G																
H																
I																
J																
K	2	0	0	0	2	1	3	1	3	1	0	1	2	0	0	0
L	1	3	3	0	0	2	2	1	2	4	9	0	3	5	3	1
M	3	0	2	0	1	2	3	3	0	3	1	0	2	0	2	1
N	2	1	0	1	0	0	3	2	2	1	0	3	1	1	4	1

	Agarose Disc (n = 19 trials)								3D Crab Shape (n = 20 trials)							
Octopus	R1	R2	R3	R4	L1	L2	L3	L4	R1	R2	R3	R4	L1	L2	L3	L4
A	2	1	2	1	4	3	4	1	3	0	0	0	3	0	0	2
B	4	6	0	5	8	6	7	5	2	3	1	0	1	0	2	1
C	0	0	0	0	2	1	3	1	1	3	3	2	2	1	2	1
D	2	5	1	4	1	1	2	1	0	1	1	0	2	1	0	0
E	2	5	1	4	1	1	2	1	4	3	4	1	2	1	4	2
F	1	4	1	1	3	5	3	5	0	1	1	3	3	0	3	0
G									1	2	1	0	1	1	2	2
H									0	0	0	0	0	0	0	1
I									2	3	1	2	1	1	4	3
J									4	2	1	1	2	3	1	1
K	3	3	3	0	4	4	4	2								
L	2	2	3	1	1	1	4	3								
M	0	1	3	1	1	0	4	3								
N	7	2	1	1	4	5	3	6								

	Live Crab (n = 20 trials)							
Octopus	R1	R2	R3	R4	L1	L2	L3	L4
A	4	1	0	0	1	0	0	0
B	0	1	0	0	0	1	2	0
C	0	1	1	0	1	0	0	0
D	2	1	0	0	2	0	1	0
E	3	8	0	1	3	2	2	3
F	2	2	2	0	2	1	0	0
G	1	2	1	1	1	3	2	2
H	4	1	0	2	2	2	2	2
I	0	2	1	2	4	2	1	2
J	2	1	0	3	4	3	3	1
K								
L								
M								
N								

Biology Open • Supplementary information

Table S5. Results of post-hoc Wilcoxon signed-rank tests with false discovery rate correction (Benjamini, Krieger and Yekutieli method) for comparisons of arm use during item contact and manipulation.

Two-stage linear step-up procedure of Benjamini, Krieger and Yekutieli	Rank sum difference	Discovery?	*q* value	Individual *p* value
R1 vs. R2	-2.00	No	0.691	0.877
R1 vs. R3	28.0	No	0.075	0.031
R1 vs. R4*	36.5	Yes	0.021	0.005
R1 vs. L1	-7.50	No	0.540	0.563
R1 vs. L2	3.50	No	0.643	0.787
R1 vs. L3	-11.0	No	0.460	0.396
R1 vs. L4	12.5	No	0.434	0.335
R2 vs. R3	30.0	No	0.057	0.021
R2 vs. R4*	38.5	Yes	0.016	0.003
R2 vs. L1	-5.50	No	0.592	0.671
R2 vs. L2	5.50	No	0.592	0.671
R2 vs. L3	-9.00	No	0.512	0.488
R2 vs. L4	14.5	No	0.363	0.263
R3 vs. R4	8.50	No	0.513	0.512
R3 vs. L1*	-35.5	Yes	0.023	0.006
R3 vs. L2	-24.5	No	0.128	0.059
R3 vs. L3*	-39.0	Yes	0.016	0.003
R3 vs. L4	-15.5	No	0.363	0.232
R4 vs. L1*	-44.0	Yes	0.008	< 0.001
R4 vs. L2*	-33.0	Yes	0.034	0.011
R4 vs. L3*	-47.5	Yes	0.006	< 0.001
R4 vs. L4	-24.0	No	0.128	0.064
L1 vs. L2	11.0	No	0.460	0.396
L1 vs. L3	-3.50	No	0.643	0.787
L1 vs. L4	20.0	No	0.208	0.113
L2 vs. L3	-14.5	No	0.363	0.263
L2 vs. L4	9.00	No	0.512	0.488
L3 vs. L4	23.5	No	0.128	0.070

Biology Open • Supplementary information

Table S6. Total observed occurrences of arm recruitments between any two arms used sequentially to contact the item inside of the dome in each trial (*n* = 117), recorded per octopus and separated by item presented. Grey cells indicate the octopus was not presented with that object. Zeros indicate true zero occurrences.

	Frozen Crab (n = 38 trials)				3D Rock Shape (n = 20 trials)			
	Recruitment Type				Recruitment Type			
Octopus	A+1	A+2	A+3	A+4	A+1	A+2	A+3	A+4
A	1	3	0	0	3	1	1	1
B	2	3	1	2	5	2	0	3
C	5	5	3	0	0	1	0	0
D	5	2	6	0	6	1	0	0
E	5	2	3	5	1	0	1	1
F	6	4	6	5	2	5	1	0
G								
H								
I								
J								
K	3	0	2	0	2	0	0	0
L	3	0	1	0	5	3	2	1
M	1	4	2	3	1	0	2	1
N	1	0	2	2	2	2	0	0

	Agarose Disc (n = 19 trials)				3D Crab Shape (n = 20 trials)			
	Recruitment Type				Recruitment Type			
Octopus	A+1	A+2	A+3	A+4	A+1	A+2	A+3	A+4
A	5	2	1	0	1	0	0	3
B	6	1	3	4	1	2	2	0
C	4	0	0	0	4	2	2	0
D	5	1	0	0	2	0	0	0
E	6	6	2	1	4	5	4	2
F	7	1	0	0	1	3	1	1
G					4	3	0	1
H					0	0	0	0
I					4	2	0	1
J					4	5	3	1
K	8	0	1	1				
L	5	2	0	2				
M	5	1	2	0				
N	7	2	1	0				

	Live Crab (n = 20 trials) Recruitment Type			
Octopus	A+1	A+2	A+3	A+4
A	1	0	0	0
B	1	0	0	0
C	1	0	0	0
D	3	0	1	0
E	4	10	4	1
F	4	1	2	0
G	4	3	2	1
H	6	4	2	1
I	5	3	2	2
J	4	3	5	0
K				
L				
M				
N				

Table S7. Total number of recruitment patterns among the first three arms used to contact the item inside of the dome in each approach of every trial where at least three different arms were used (*n* = 83 sequence patterns) for all octopuses (*n* = 14).

		Recruitment type between initial contacting arm and second contacting arm			
		A+1	A+2	A+3	A+4
Recruitment type between second arm and third contacting arm	A+1	22	7	2	2
	A+2	11	5	3	2
	A+3	7	4	3	3
	A+4	4	3	4	1

Table S8. Total occurrences of the maximum number of arms observed simultaneously inside of the dome used for exploring and contacting in each trial (n = 117), recorded per octopus.

	Number of arms							
Octopus	1	2	3	4	5	6	7	8
A	0	1	8	2	1	0	0	0
B	1	1	5	3	2	0	0	0
C	0	0	2	4	1	2	0	0
D	0	0	1	4	6	1	0	0
E	0	1	3	1	7	0	0	0
F	0	0	1	4	5	1	1	0
G	0	0	1	1	2	0	0	0
H	0	1	0	2	1	0	0	0
I	0	0	0	2	2	0	0	0
J	0	0	0	2	2	0	0	0
K	0	0	5	3	0	0	0	0
L	0	0	0	4	4	0	0	0
M	0	1	1	3	2	1	0	0
N	0	1	5	1	1	0	0	0

Table S9. Origin, wet weight, and total number of trials per item for each octopus.

			Total number of experimental trials				
Octopus	Origin	Wet Weight (g)	Frozen Crab	3D Rock Shape	Agarose Disc	3D Crab Shape	Live Crab
A	Wild-caught	190.03	4	2	2	2	2
B	Wild-caught	209.04	4	2	2	2	2
C	Lab-reared	192.21	2	2	1	2	2
D	Lab-reared	181.61	4	2	2	2	2
E	Lab-reared	115.96	4	2	2	2	2
F	Lab-reared	74.32	4	2	2	2	2
G	Lab-reared	79.85	0	0	0	2	2
H	Lab-reared	141.13	0	0	0	2	2
I	Lab-reared	125.59	0	0	0	2	2
J	Lab-reared	153.88	0	0	0	2	2
K	Wild-caught	216.82	4	2	2	0	0
L	Wild-caught	248.55	4	2	2	0	0
M	Wild-caught	332.24	4	2	2	0	0
N	Wild-caught	254.75	4	2	2	0	0

Biology Open • Supplementary information
